# 6,8-Dihy­droxy-8a-methyl-3,5-dimethyl­idenedeca­hydro­naphtho­[2,3-*b*]furan-2(3*H*)-one

**DOI:** 10.1107/S160053681204425X

**Published:** 2012-10-31

**Authors:** Ting-ting Liu, Hai-Bo Wu, Wen-Shu Wang

**Affiliations:** a College of Life and Environment Science, Minzu University of China, Beijing 100081, People’s Republic of China

## Abstract

The title compound, C_15_H_20_O_4_, is a eudesmanolide isolated from the Chinese medicinal plant *Carpesium tris­te* Maxim. The mol­ecule contains three rings, *viz.* two fused six-membered rings in chair conformations and a five-membered ring in a flattened envelope conformation. In the crystal, two hy­droxy groups are involved in the formation of intra- and inter­molecular O—H⋯O hydrogen bonds. The H atoms in these groups are split, with site-occupation factors of 0.5. The inter­molecular hydrogen bonds link mol­ecules into chains propagating in [010].

## Related literature

For related compounds extracted from *Carpesium tris­te* Maxim, see: Masao & Fumiko (1975 ▶).
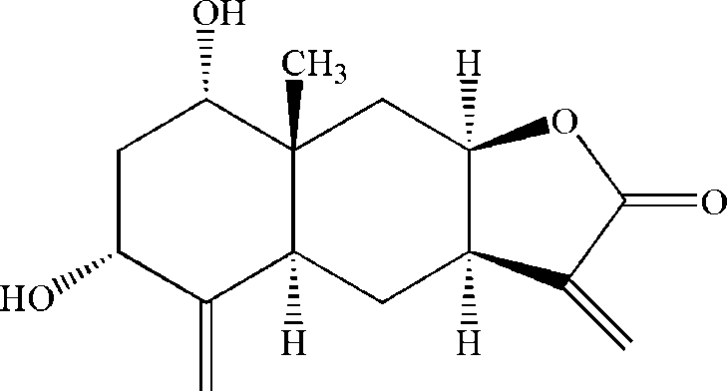


## Experimental

### 

#### Crystal data

C_15_H_20_O_4_
*M* *_r_* = 264.31
Tetragonal, 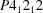

*a* = 6.4737 (4) Å
*c* = 62.438 (8) Å
*V* = 2616.7 (5) Å^3^
*Z* = 8
Mo *K*α radiation
μ = 0.10 mm^−1^
*T* = 133 K
0.54 × 0.48 × 0.32 mm

#### Data collection

Rigaku AFC10/Saturn-724+ CCD diffractometer
19607 measured reflections
1990 independent reflections
1826 reflections with *I* > 2σ(*I*)
*R*_int_ = 0.058

#### Refinement

*R*[*F* ^2^ > 2σ(*F* ^2^)] = 0.047
*wR*(*F* ^2^) = 0.124
*S* = 1.00
1990 reflections
185 parameters
6 restraints
H atoms treated by a mixture of independent and constrained refinement
Δρ_max_ = 0.23 e Å^−3^
Δρ_min_ = −0.21 e Å^−3^

### 

Data collection: *CrystalClear* (Rigaku/MSC, 2008 ▶); cell refinement: *CrystalClear*; data reduction: *CrystalClear*; program(s) used to solve structure: *SHELXS97* (Sheldrick, 2008 ▶); program(s) used to refine structure: *SHELXL97* (Sheldrick, 2008 ▶); molecular graphics: *SHELXTL* (Sheldrick, 2008 ▶); software used to prepare material for publication: *SHELXTL*.

## Supplementary Material

Crystal structure: contains datablock(s) I, global. DOI: 10.1107/S160053681204425X/rk2376sup1.cif

Structure factors: contains datablock(s) I. DOI: 10.1107/S160053681204425X/rk2376Isup2.hkl

Supplementary material file. DOI: 10.1107/S160053681204425X/rk2376Isup3.cml

Additional supplementary materials:  crystallographic information; 3D view; checkCIF report

## Figures and Tables

**Table 1:** Hydrogen-bond geometry (Å, °)

*D*—H⋯*A*	*D*—H	H⋯*A*	*D*⋯*A*	*D*—H⋯*A*
O2—H2*O*⋯O2^i^	0.84 (1)	1.90 (1)	2.733 (3)	173 (5)
O2—H2*O*′⋯O3	0.84 (1)	1.94 (2)	2.690 (2)	148 (2)
O3—H3*O*′⋯O2	0.84 (1)	1.97 (4)	2.690 (2)	143 (5)
O3—H3*O*⋯O3^ii^	0.83 (1)	1.88 (2)	2.697 (3)	168 (6)

Symmetry codes: (i) 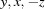; (ii) 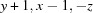.

**Fig. 1.**
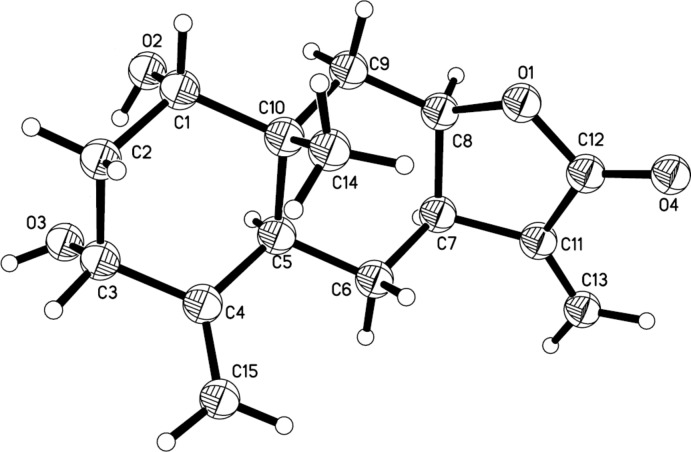
View of the title molecule showing the atom-numbering scheme. Displacement ellipsoids are drawn at the 50% probability level. H atoms are presented as a small spheres of arbitrary radius. In the both hydroxy groups only one H atom is presented.

## supplementary crystallographic information

### Comment

*Carpesium triste* Maxim grows in the northeast and southwest area of mainland China. It is used as traditional Chinese medicine having effects on detoxification and antibacterial activities. As a part of our research on biological resource by ethnic minorities in Guizhou province, the title compound was isolated form *Carpesium triste* Maxim. Its structure was identified by NMR spectra data and compared with the previous reports (Masao & Fumiko, 1975). Herewith we present its molecular structure. The molecule of the title compound contains a three-ring system *A*/*B*/*C* (Fig. 1). Ring *A* and *B* are both in chair conformations and there is a *trans*-junction between ring *A* (C1-C5/C10) and ring *B* (C5-C7/C8-C10). Furthermore, the methyl group at C14 sites is in the opposite orientation with the two hydroxyl groups at C1 and C3 sites. The furan ring *C* (C8-C12/O1) is in an envelope-like conformation. Two hydroxy groups contribute to the formation of intra- and inter-molecular O–H···O hydrogen bonds (Table 1). The latter ones link molecules into chain propagated in direction [0 1 0].

### Experimental

The air-dried whole plant of *Carpesium triste* Maxim (0.337 kg) were pulverized and extracted three times with CH_3_OH (each for less than 1 minutes) at room temperature by flash-type extractor. The extract was concentrated to give a residue (33.5 g), which was further separated by *CC* (SiO_2_, 200-300 mesh, petroleum ether/acetone (25:1, 20:1, 15:1, 10:1, 8:1, 5:1, 3:1, 2:1, 1:1(*v*/*v*)) to yield 9 fractions: Fr. 1-9. Each fraction was examined by *TLC* and combined to afford many subfractions. Fr. 5 (270 mg) was subjected to Sephadex LH-20 (CHCl_3_/CH_3_OH 1:1) to provide the title compound (4 mg). ^1^H and ^13^C NMR spectral data of this compound was recorded on Bruker-AV-500 spectrometer, using CDCl_3_ as solvent and *Me*_4_Si as internal standard. The relative stereochemistry can be observed by X-ray diffraction experiment.

### Refinement

The hydrogen atoms which bonded with C were placed in calculated positions with C–H = 0.95-1.00Å. The hydroxyl H atoms were located in Fourier difference map. The H atoms in these droups are splitted with s.o.f. = 0.5. The positions of hydroxyl H atoms were refined freely. All H atoms were refined with *U*_iso_(H) = 1.2*U*_eq_(C, O).

### Crystal data

**Table:**

C_15_H_20_O_4_	*D*_x_ = 1.342 Mg m^−^^3^
*M**_r_* = 264.31	Mo *K*α radiation, λ = 0.71073 Å
Tetragonal, *P*4_1_2_1_2	Cell parameters from 4658 reflections
Hall symbol: P 4abw 2nw	θ = 3.2–27.9°
*a* = 6.4737 (4) Å	µ = 0.10 mm^−^^1^
*c* = 62.438 (8) Å	*T* = 133 K
*V* = 2616.7 (5) Å^3^	Block, colourless
*Z* = 8	0.54 × 0.48 × 0.32 mm
*F*(000) = 1136	

### Data collection

**Table:**

Rigaku AFC10/Saturn-724+ CCD diffractometer	1826 reflections with *I* > 2σ(*I*)
Radiation source: Rotating Anode	*R*_int_ = 0.058
Graphite monochromator	θ_max_ = 27.9°, θ_min_ = 2.6°
Detector resolution: 28.5714 pixels mm^-1^	*h* = −8→8
φ– and ω–scans	*k* = −8→8
19607 measured reflections	*l* = −82→82
1990 independent reflections	

### Refinement

**Table:**

Refinement on *F*^2^	Primary atom site location: structure-invariant direct methods
Least-squares matrix: full	Secondary atom site location: difference Fourier map
*R*[*F*^2^ > 2σ(*F*^2^)] = 0.047	Hydrogen site location: inferred from neighbouring sites
*wR*(*F*^2^) = 0.124	H atoms treated by a mixture of independent and constrained refinement
*S* = 1.00	*w* = 1/[σ^2^(*F*_o_^2^) + (0.0836*P*)^2^ + 0.316*P*] where *P* = (*F*_o_^2^ + 2*F*_c_^2^)/3
1990 reflections	(Δ/σ)_max_ = 0.001
185 parameters	Δρ_max_ = 0.23 e Å^−^^3^
6 restraints	Δρ_min_ = −0.21 e Å^−^^3^

### Special details

**Table:**

Experimental. Since the skeleton methyl groups in eudesmanolide are biogenic 8a position, we draw the relative stereochemistry of the title eudesmanolide, by reference to the structures of related eudesmanolide in (Masao & Fumiko, 1975) although the absolute configuration could not be reliably determined from anomalous dispersion effects, if Mo radiation is used in experiment. Furthermore, the relative stereochemistry in the title compound was confirmed by NMR data. ^13^C NMR (125 MHz, CDCl_3_, δ,p.p.m.): 178.1 (C12), 149.2 (C4), 141.4 (C11), 120.5 (C13), 110.9 (C15), 77.6 (C8), 75.2 (C3), 74.7 (C1), 63.8 (C10), 40.3 (C7), 33.9 (C5), 33.6 (C9), 33.5 (C2), 26.8 (C6), 17.7 (C14).
Geometry. All s.u.'s (except the s.u. in the dihedral angle between two l.s. planes) are estimated using the full covariance matrix. The cell s.u.'s are taken into account individually in the estimation of s.u.'s in distances, angles and torsion angles; correlations between s.u.'s in cell parameters are only used when they are defined by crystal symmetry. An approximate (isotropic) treatment of cell s.u.'s is used for estimating s.u.'s involving l.s. planes.
Refinement. Refinement of *F*^2^ against ALL reflections. The weighted *R*-factor *wR* and goodness of fit *S* are based on *F*^2^, conventional *R*-factors *R* are based on *F*, with *F* set to zero for negative *F*^2^. The threshold expression of *F*^2^ > σ(*F*^2^) is used only for calculating *R*-factors(gt) *etc*. and is not relevant to the choice of reflections for refinement. *R*-factors based on *F*^2^ are statistically about twice as large as those based on *F*, and *R*-factors based on ALL data will be even larger.

### Fractional atomic coordinates and isotropic or equivalent isotropic displacement parameters (Å^2^)

**Table:**

	*x*	*y*	*z*	*U*_iso_*/*U*_eq_	Occ. (<1)
O1	0.6749 (2)	0.7974 (2)	0.09621 (2)	0.0238 (3)	
O2	0.9609 (2)	0.7729 (3)	0.01700 (2)	0.0259 (4)	
H2O	0.903 (5)	0.820 (6)	0.0061 (5)	0.031*	0.50
H2O'	1.080 (3)	0.727 (4)	0.0149 (8)	0.031*	0.50
O3	1.2633 (3)	0.4894 (3)	0.01385 (3)	0.0277 (4)	
H3O	1.329 (7)	0.432 (8)	0.0040 (7)	0.033*	0.50
H3O'	1.216 (8)	0.609 (4)	0.0132 (9)	0.033*	0.50
O4	0.6433 (3)	0.7218 (3)	0.13102 (3)	0.0289 (4)	
C1	0.8323 (3)	0.6114 (3)	0.02599 (3)	0.0222 (4)	
H1	0.6856	0.6382	0.0218	0.027*	
C2	0.8995 (3)	0.4040 (4)	0.01641 (3)	0.0252 (5)	
H2A	0.8023	0.2947	0.0210	0.030*	
H2B	0.8947	0.4124	0.0006	0.030*	
C3	1.1183 (3)	0.3474 (3)	0.02353 (3)	0.0230 (4)	
H3	1.1504	0.2041	0.0186	0.028*	
C4	1.1394 (3)	0.3561 (3)	0.04749 (3)	0.0188 (4)	
C5	1.0717 (3)	0.5576 (3)	0.05753 (3)	0.0176 (4)	
H5	1.1636	0.6681	0.0517	0.021*	
C6	1.0932 (3)	0.5634 (3)	0.08181 (3)	0.0197 (4)	
H6A	1.2369	0.5273	0.0857	0.024*	
H6B	1.0010	0.4578	0.0881	0.024*	
C7	1.0399 (3)	0.7753 (3)	0.09141 (3)	0.0199 (4)	
H7	1.1590	0.8724	0.0898	0.024*	
C8	0.8417 (3)	0.8722 (3)	0.08222 (3)	0.0206 (4)	
H8	0.8518	1.0254	0.0840	0.025*	
C9	0.7925 (3)	0.8280 (3)	0.05891 (3)	0.0208 (4)	
H9A	0.8663	0.9307	0.0500	0.025*	
H9B	0.6427	0.8502	0.0567	0.025*	
C10	0.8472 (3)	0.6116 (3)	0.05061 (3)	0.0196 (4)	
C11	0.9826 (3)	0.7535 (3)	0.11464 (3)	0.0221 (4)	
C12	0.7532 (3)	0.7548 (3)	0.11580 (3)	0.0217 (4)	
C13	1.1009 (4)	0.7239 (4)	0.13160 (3)	0.0287 (5)	
H13A	1.0399	0.7022	0.1453	0.034*	
H13B	1.2470	0.7243	0.1301	0.034*	
C14	0.6907 (3)	0.4523 (3)	0.05931 (4)	0.0235 (5)	
H14A	0.6728	0.4728	0.0747	0.028*	
H14B	0.7424	0.3124	0.0566	0.028*	
H14C	0.5577	0.4707	0.0521	0.028*	
C15	1.2136 (3)	0.1949 (3)	0.05820 (4)	0.0253 (5)	
H15A	1.2536	0.0735	0.0507	0.030*	
H15B	1.2267	0.2010	0.0733	0.030*	

### Atomic displacement parameters (Å^2^)

**Table:**

	*U*^11^	*U*^22^	*U*^33^	*U*^12^	*U*^13^	*U*^23^
O1	0.0227 (8)	0.0298 (8)	0.0189 (7)	0.0009 (6)	0.0021 (6)	0.0013 (6)
O2	0.0313 (9)	0.0272 (8)	0.0192 (8)	−0.0013 (7)	0.0007 (6)	0.0057 (6)
O3	0.0311 (9)	0.0287 (8)	0.0232 (8)	−0.0024 (7)	0.0085 (7)	0.0027 (7)
O4	0.0321 (9)	0.0319 (8)	0.0228 (8)	0.0021 (7)	0.0073 (7)	0.0024 (7)
C1	0.0221 (10)	0.0250 (10)	0.0196 (10)	−0.0023 (8)	−0.0023 (8)	0.0024 (8)
C2	0.0281 (11)	0.0314 (12)	0.0161 (10)	−0.0054 (9)	−0.0024 (8)	−0.0028 (9)
C3	0.0272 (11)	0.0210 (10)	0.0208 (10)	−0.0026 (8)	0.0038 (9)	−0.0021 (8)
C4	0.0183 (9)	0.0207 (9)	0.0173 (10)	−0.0031 (7)	0.0014 (8)	−0.0015 (8)
C5	0.0182 (9)	0.0187 (10)	0.0158 (9)	−0.0015 (7)	−0.0012 (7)	−0.0003 (8)
C6	0.0215 (10)	0.0211 (10)	0.0164 (9)	0.0004 (8)	−0.0008 (8)	0.0014 (8)
C7	0.0213 (10)	0.0212 (10)	0.0171 (9)	−0.0017 (8)	−0.0012 (8)	0.0010 (8)
C8	0.0241 (10)	0.0198 (9)	0.0178 (10)	−0.0008 (8)	0.0015 (8)	0.0009 (8)
C9	0.0222 (10)	0.0212 (10)	0.0190 (10)	0.0024 (8)	−0.0002 (8)	0.0025 (8)
C10	0.0183 (9)	0.0234 (10)	0.0169 (10)	−0.0012 (8)	−0.0011 (8)	0.0017 (8)
C11	0.0265 (10)	0.0202 (10)	0.0197 (10)	−0.0004 (8)	0.0014 (8)	−0.0010 (8)
C12	0.0282 (11)	0.0201 (10)	0.0169 (9)	0.0007 (8)	0.0015 (8)	−0.0007 (8)
C13	0.0318 (12)	0.0360 (12)	0.0182 (10)	−0.0009 (10)	−0.0014 (9)	0.0003 (9)
C14	0.0203 (10)	0.0257 (11)	0.0245 (10)	−0.0056 (8)	0.0015 (8)	0.0005 (9)
C15	0.0256 (10)	0.0246 (11)	0.0255 (11)	−0.0009 (8)	0.0008 (9)	−0.0012 (8)

### Geometric parameters (Å, º)

**Table:**

O1—C12	1.353 (3)	C6—C7	1.536 (3)
O1—C8	1.471 (2)	C6—H6A	0.9900
O2—C1	1.449 (3)	C6—H6B	0.9900
O2—H2O	0.840 (10)	C7—C11	1.504 (3)
O2—H2O'	0.839 (10)	C7—C8	1.539 (3)
O3—C3	1.447 (3)	C7—H7	1.0000
O3—H3O	0.834 (10)	C8—C9	1.517 (3)
O3—H3O'	0.836 (10)	C8—H8	1.0000
O4—C12	1.206 (3)	C9—C10	1.535 (3)
C1—C2	1.533 (3)	C9—H9A	0.9900
C1—C10	1.540 (3)	C9—H9B	0.9900
C1—H1	1.0000	C10—C14	1.544 (3)
C2—C3	1.529 (3)	C11—C13	1.321 (3)
C2—H2A	0.9900	C11—C12	1.486 (3)
C2—H2B	0.9900	C13—H13A	0.9500
C3—C4	1.503 (3)	C13—H13B	0.9500
C3—H3	1.0000	C14—H14A	0.9800
C4—C15	1.330 (3)	C14—H14B	0.9800
C4—C5	1.512 (3)	C14—H14C	0.9800
C5—C6	1.523 (3)	C15—H15A	0.9500
C5—C10	1.556 (3)	C15—H15B	0.9500
C5—H5	1.0000		
			
C12—O1—C8	109.21 (16)	C11—C7—C8	101.06 (17)
C1—O2—H2O	108.7 (12)	C6—C7—C8	113.93 (16)
C1—O2—H2O'	109.6 (12)	C11—C7—H7	110.4
H2O—O2—H2O'	115 (5)	C6—C7—H7	110.4
C3—O3—H3O	111 (4)	C8—C7—H7	110.4
C3—O3—H3O'	112 (4)	O1—C8—C9	110.70 (17)
H3O—O3—H3O'	124 (6)	O1—C8—C7	104.87 (15)
O2—C1—C2	108.53 (17)	C9—C8—C7	117.13 (17)
O2—C1—C10	110.48 (16)	O1—C8—H8	107.9
C2—C1—C10	111.86 (17)	C9—C8—H8	107.9
O2—C1—H1	108.6	C7—C8—H8	107.9
C2—C1—H1	108.6	C8—C9—C10	116.56 (16)
C10—C1—H1	108.6	C8—C9—H9A	108.2
C3—C2—C1	111.05 (17)	C10—C9—H9A	108.2
C3—C2—H2A	109.4	C8—C9—H9B	108.2
C1—C2—H2A	109.4	C10—C9—H9B	108.2
C3—C2—H2B	109.4	H9A—C9—H9B	107.3
C1—C2—H2B	109.4	C9—C10—C1	108.84 (16)
H2A—C2—H2B	108.0	C9—C10—C14	109.84 (17)
O3—C3—C4	109.47 (17)	C1—C10—C14	108.02 (16)
O3—C3—C2	109.10 (17)	C9—C10—C5	109.08 (16)
C4—C3—C2	111.38 (17)	C1—C10—C5	109.60 (16)
O3—C3—H3	108.9	C14—C10—C5	111.41 (17)
C4—C3—H3	108.9	C13—C11—C12	122.7 (2)
C2—C3—H3	108.9	C13—C11—C7	130.1 (2)
C15—C4—C3	120.26 (19)	C12—C11—C7	107.06 (18)
C15—C4—C5	124.99 (19)	O4—C12—O1	121.8 (2)
C3—C4—C5	114.75 (17)	O4—C12—C11	128.8 (2)
C4—C5—C6	114.06 (17)	O1—C12—C11	109.35 (17)
C4—C5—C10	110.44 (16)	C11—C13—H13A	120.0
C6—C5—C10	110.86 (16)	C11—C13—H13B	120.0
C4—C5—H5	107.0	H13A—C13—H13B	120.0
C6—C5—H5	107.0	C10—C14—H14A	109.5
C10—C5—H5	107.0	C10—C14—H14B	109.5
C5—C6—C7	112.94 (16)	H14A—C14—H14B	109.5
C5—C6—H6A	109.0	C10—C14—H14C	109.5
C7—C6—H6A	109.0	H14A—C14—H14C	109.5
C5—C6—H6B	109.0	H14B—C14—H14C	109.5
C7—C6—H6B	109.0	C4—C15—H15A	120.0
H6A—C6—H6B	107.8	C4—C15—H15B	120.0
C11—C7—C6	110.34 (17)	H15A—C15—H15B	120.0
			
O2—C1—C2—C3	65.9 (2)	C8—C9—C10—C14	74.5 (2)
C10—C1—C2—C3	−56.2 (2)	C8—C9—C10—C5	−47.9 (2)
C1—C2—C3—O3	−68.2 (2)	O2—C1—C10—C9	55.0 (2)
C1—C2—C3—C4	52.7 (2)	C2—C1—C10—C9	176.05 (17)
O3—C3—C4—C15	−112.1 (2)	O2—C1—C10—C14	174.25 (17)
C2—C3—C4—C15	127.1 (2)	C2—C1—C10—C14	−64.7 (2)
O3—C3—C4—C5	67.6 (2)	O2—C1—C10—C5	−64.2 (2)
C2—C3—C4—C5	−53.2 (2)	C2—C1—C10—C5	56.8 (2)
C15—C4—C5—C6	−0.6 (3)	C4—C5—C10—C9	−173.51 (16)
C3—C4—C5—C6	179.74 (17)	C6—C5—C10—C9	59.1 (2)
C15—C4—C5—C10	−126.2 (2)	C4—C5—C10—C1	−54.4 (2)
C3—C4—C5—C10	54.1 (2)	C6—C5—C10—C1	178.14 (16)
C4—C5—C6—C7	175.41 (16)	C4—C5—C10—C14	65.1 (2)
C10—C5—C6—C7	−59.2 (2)	C6—C5—C10—C14	−62.4 (2)
C5—C6—C7—C11	157.85 (17)	C6—C7—C11—C13	76.2 (3)
C5—C6—C7—C8	45.0 (2)	C8—C7—C11—C13	−162.9 (2)
C12—O1—C8—C9	153.66 (17)	C6—C7—C11—C12	−99.4 (2)
C12—O1—C8—C7	26.4 (2)	C8—C7—C11—C12	21.5 (2)
C11—C7—C8—O1	−28.42 (19)	C8—O1—C12—O4	168.33 (19)
C6—C7—C8—O1	89.89 (19)	C8—O1—C12—C11	−12.6 (2)
C11—C7—C8—C9	−151.58 (17)	C13—C11—C12—O4	−3.7 (4)
C6—C7—C8—C9	−33.3 (2)	C7—C11—C12—O4	172.3 (2)
O1—C8—C9—C10	−83.9 (2)	C13—C11—C12—O1	177.4 (2)
C7—C8—C9—C10	36.2 (3)	C7—C11—C12—O1	−6.6 (2)
C8—C9—C10—C1	−167.46 (17)		

### Hydrogen-bond geometry (Å, º)

**Table:**

*D*—H···*A*	*D*—H	H···*A*	*D*···*A*	*D*—H···*A*
O2—H2*O*···O2^i^	0.84 (1)	1.90 (1)	2.733 (3)	173 (5)
O2—H2*O*′···O3	0.84 (1)	1.94 (2)	2.690 (2)	148 (2)
O3—H3*O*′···O2	0.84 (1)	1.97 (4)	2.690 (2)	143 (5)
O3—H3*O*···O3^ii^	0.83 (1)	1.88 (2)	2.697 (3)	168 (6)

Symmetry codes: (i) *y*, *x*, −*z*; (ii) *y*+1, *x*−1, −*z*.
